# A Randomized Pilot Study of DASH Patterned Groceries on Serum Urate in Individuals with Gout

**DOI:** 10.3390/nu13020538

**Published:** 2021-02-07

**Authors:** Stephen P. Juraschek, Edgar R. Miller, Beiwen Wu, Karen White, Jeanne Charleston, Allan C. Gelber, Sharan K. Rai, Kathryn A. Carson, Lawrence J. Appel, Hyon K. Choi

**Affiliations:** 1 Beth Israel Deaconess Medical Center, General Medicine, Boston, MA 02215, USA; 2 Johns Hopkins Bloomberg School of Public Health, Welch Center for Prevention, Epidemiology and Clinical Research, Johns Hopkins University School of Medicine, Baltimore, MD 21205, USA; ermiller@jhmi.edu (E.R.M.III); bwu19@jhmi.edu (B.W.); kwhite33@jhmi.edu (K.W.); jeannec@jhmi.edu (J.C.); agelber@jhmi.edu (A.C.G.); kcarson@jhmi.edu (K.A.C.); lappel@jhmi.edu (L.J.A.); 3 Department of Nutrition, Harvard T.H. Chan School of Public Health, Boston, MA 02215, USA; shr820@g.harvard.edu; 4 Division of Rheumatology, Allergy, and Immunology, Massachusetts General Hospital, Harvard Medical School, Boston, MA 02215, USA; hchoi@mgh.harvard.edu

**Keywords:** diet, serum urate, gout, hypertension

## Abstract

The Dietary Approaches to Stop Hypertension (DASH) diet reduces serum urate (SU); however, the impact of the DASH diet has not been previously evaluated among patients with gout. We conducted a randomized, controlled, crossover pilot study to test the effects of ~$105/week ($15/day) of dietitian-directed groceries (DDG), patterned after the DASH diet, on SU, compared with self-directed grocery shopping (SDG). Participants had gout and were not taking urate lowering therapy. Each intervention period lasted 4 weeks; crossover occurred without a washout period. The primary endpoint was SU. Compliance was assessed by end-of-period fasting spot urine potassium and sodium measurements and self-reported consumption of daily servings of fruit and vegetables. We randomized 43 participants (19% women, 49% black, mean age 59 years) with 100% follow-up. Mean baseline SU was 8.1 mg/dL (SD, 0.8). During Period 1, DDG lowered SU by 0.55 mg/dL (95% CI: 0.07, 1.04) compared to SDG by 0.0 mg/dL (95% CI: −0.44, 0.44). However, after crossover (Period 2), the SU difference between groups was the opposite: SDG reduced SU by −0.48 mg/dL (95% CI: −0.98, 0.01) compared to DDG by −0.05 mg/dL (95% CI: −0.48, 0.38; P for interaction by period = 0.11). Nevertheless, DDG improved self-reported intake of fruit and vegetables (3.1 servings/day; 95% CI: 1.5, 4.8) and significantly reduced total spot urine sodium excretion by 22 percentage points (95% CI: −34.0, −8.6). Though relatively small in scale, this pilot study suggests that dietitian-directed, DASH-patterned groceries may lower SU among gout patients not on urate-lowering drugs. However, behavior intervention crossover trials without a washout period are likely vulnerable to strong carryover effects. Definitive evaluation of the DASH diet as a treatment for gout will require a controlled feeding trial, ideally with a parallel-design.

## 1. Introduction

The disease burden of gout has been increasing worldwide, a trend thought to be driven by concurrent epidemics in obesity and Western lifestyle [1,2,3,4]. Interventions that can effectively reduce serum urate (SU) while simultaneously improving cardiovascular risk factors are critically needed to prevent and treat gout comprehensively.

Diet has long been implicated in the pathogenesis of gout, including a higher intake of meat, seafood, sugar-sweetened beverages, and overall Western-dietary pattern [5,6,7,8]. Several studies have identified specific elements of diet, such as vitamin C [9,10] or dairy products, [11] that lower SU. More recently, the Dietary Approaches to Stop Hypertension (DASH) diet, a dietary pattern emphasizing fruit, vegetables, and low-fat dairy products that is proven to reduce blood pressure and low density lipoprotein cholesterol (LDL-cholesterol), [12,13] has been associated with a lower risk of gout among men, [14] and has been shown to lower SU by 0.35 mg/dL overall among those with a SU level of 5 mg/dL at baseline and by over 1 mg/dL in those with an elevated SU [15,16]. However, whether a DASH-patterned diet improves SU in gout patients has not been previously demonstrated.

The objective of the present study was to conduct a pilot study testing whether partial replacement of diet with the provision of DASH-patterned groceries over a 4-week period would lower SU level. 

## 2. Methods

The Dietary Approaches to Stop Hypertension Diet Effects on Serum Uric Acid (SU) in Adults with Hyperuricemia and Gout (DIGO) was an investigator-initiated, randomized crossover trial conducted in Baltimore, Maryland. The study was sponsored by the Rheumatology Research Foundation. The foundation did not play any role in the design or conduct of the study beyond its funding. DIGO was a pilot study that examined the effect of a 4-week, weekly provision of $105 of groceries patterned after the DASH diet on SU levels. Each participant provided written informed consent. The first contact for recruitment occurred on 20 June 2018. The first participant was enrolled (randomized) on 7 August 2018. Data collection ended 23 July 2019. All participants provided written, informed consent to participate in this trial. The study was conducted in accordance with the Declaration of Helsinki and received ethical approval from the Institutional Review Board (IRB) at Johns Hopkins University School of Medicine (IRB00153409).

### 2.1. Participants

Eligible participants were community-dwelling adults, aged ≥18 years, with a self-reported diagnosis of gout and a SU concentration ≥7 mg/dL. Gout was based on self-report in response to the question “Has a physician told you that you have gout?” Exclusion criteria included: active use of or plans for urate lowering therapy, excessive alcohol use, stage 4 or 5 chronic kidney disease, unstable medication use (steroid, lipid-lowering, or antihypertensive agents), active prescriptions of warfarin or insulin, major gastrointestinal conditions affecting food absorption, or inability to store food at home. 

Participants were recruited by identifying patients with a diagnosis of gout in the Johns Hopkins Medicine medical record, newspaper advertisements, Facebook advertisements, and mass mailings to adults living in the communities surrounding the research center [17]. After completion of two in-person visits at the Johns Hopkins ProHealth Clinical Research Unit (Woodlawn, Maryland, USA), eligible adults were randomized to one of two 4-week intervention sequences: the provision of DASH-patterned groceries followed by self-directed grocery purchases or self-directed grocery purchases followed by the provision of DASH-patterned groceries. These two interventions were not separated by a washout period. Each period concluded with a follow-up visit, which included SU measurement.

### 2.2. Intervention

DIGO tested the provision of DASH-patterned groceries ordered with the assistance of a dietitian (i.e., dietitian-directed or DDG) on urate levels compared to self-directed grocery shopping (SDG). During the DDG intervention, participants participated in a one-on-one session with a dietitian at the initiation of the intervention, followed by weekly phone calls thereafter. The educational content of these sessions was restricted to instructions to eat the study foods and avoid non-study foods (see Supplement Materials S1–S3). SDG were asked to continue their typical dietary habits. DIGO did not include a washout period between interventions as SDG was inherently a delay of the intervention (SDG followed by DDG) or a washout of intervention (DDG followed by SDG). This design decision was based on the observation that reversible dietary effects occurred within 4-week periods in prior controlled-feeding studies [15].

The initial visit lasted one hour, after which, the dietitian spoke with participants by phone each week to order the following week’s groceries. During the DDG assignment, participants were allotted a stipend of $105/week for the purchase of food (i.e., $15/day). We primarily used AmazonFresh (Seattle, Washington, USA) to order and deliver foods to the ProHealth research center for weekly pick-up by the participant. In contrast, during the SDG assignment, a grocery stipend was not provided. 

Groceries were ordered by food groups in fixed proportions reflecting the DASH diet [12] (Table 1): 5–7 servings/day of grains, 4 servings/day of fruit, 4 servings/day of vegetables, 1–2 servings/day of lean meat (poultry/fish), 2 servings/day of low fat dairy, and <0.5 servings/day of high fiber foods (nuts, seeds, legumes). During the DDG period, participants were also asked to restrict alcohol, sugar-sweetened beverages (no soda, no juice), sweets, red meat, organ meats, and shellfish. Food orders were selected to be low in fat, saturated fat, and cholesterol, consistent with the original DASH diet [12]. We also focused on groceries consistent with consuming less than 2300 mg of sodium a day. 

All AmazonFresh orders were received by DIGO personnel and examined by the study dietitians to assess for spoilage, missing or mistaken items, or damaged packaging. Overall food quality was good and orders were quite accurate (see Supplement Material S4 for qualitative notes). During the DDG period, participants were asked to use the groceries purchased through the study as the basis for their meals. Compliance with the intervention was assessed weekly during food ordering. Participants were asked to quantify how much, and which components, of the groceries they consumed.

A variable 2 or 4 block size randomization scheme was generated by a biostatistician at the Johns Hopkins Institute for Clinical and Translational Research. The scheme entailed two orders AB or BA consistent with our crossover design. Details of the block sizes were concealed from study staff and investigators, and randomization assignments generated prior to the study were placed in opaque, sealed envelopes. After confirmation of eligibility and completion of informed consent, a designated staff member external to DIGO operations opened the envelope with the participant during the randomization visit. Once the assigned order was revealed, this information was shared with the study dietitian, who provided instructions pertinent for each intervention period. Order of the intervention was not shared with research staff performing outcome assessments or with study investigators. During the trial, investigators and research staff were masked to outcome assessments.

### 2.3. Primary Outcome: Serum Urate

The primary outcome measure was fasting SU measured after each 4-week intervention period. After fasting, serum specimens were collected in a serum-separator tube, allowed to clot for 30 min, and then centrifuged for 15 min. SU quantification was performed by Quest Diagnostics (Madison, New Jersey, USA) via spectrophotometry. Specimens did not undergo freezing or storage.

### 2.4. Adherence

For all participants, adherence to dietary changes was assessed using self-reported and validated screening questionnaires for fruit, vegetable, and fat intake [18]. Respondents were asked about how often they ate a variety of foods over the prior month. These responses were used to estimate daily intake of fat, saturated fat, total fat, cholesterol, and fruits and vegetables. These brief screening tools are useful for monitoring diets and correlate well (r = 0.6 to 0.7, *p* < 0.0001) with the Block full length food frequency questionnaire [19].

Objective measures of adherence were spot urine potassium and sodium concentrations measured in urine, collected pre-randomization and after each intervention period. Urine measures were collected at the time overnight fasting blood was collected, reflecting a fasting second void. Our primary objective compliance measurement was urine potassium excretion. Electrolytes were measured via ion selective electrode by Quest Diagnostics (Madison, New Jersey, USA). We also measured urine urate concentration and pH to assess for mechanisms of SU reduction.

### 2.5. Other Measurements

#### 2.5.1. Gout-Related Outcomes

After each intervention period, participants were asked about gout-related symptoms. Following the definition described by Gaffo et al., [20] a gout flare was considered present if gout pain was associated with at least 3 of the following: at least one swollen joint, at least one warm joint, use of rescue medications to treat pain, seeking medical care to treat pain, or overnight hospitalization related to pain. Participants were also asked to rate on a 0–9 scale their pain with the following activities (a subset of questions in the Western Ontario and McMaster Universities Osteoarthritis [WOMAC] Index): walking, stair climbing, at night, at rest, or with weight bearing [21]. In addition, participants rated their bodily pain during the past 4 weeks and pain interfering with normal work (work outside the home and housework).

#### 2.5.2. Anthropometric and Laboratory Outcomes

The following assessments were performed prior to randomization and after each intervention period. We determined the average of 3 seated blood pressure measures using an automated sphygmomanometer (Omron Hem-907XL, Omron, Kyoto, Japan) after 5 min of seated rest with an interval of 30 s between measurements. Body mass index (BMI) was derived from a calibrated scale (weight) and stadiometer (height). Further, we recorded the time required for a participant to get up from a seated position, walk 3 m in a straight line, and return to the seated position (a Timed-Up-and-Go [TUG] test) [22]. 

A fasting lipid panel (HDL-cholesterol, LDL-cholesterol, triglycerides, and total cholesterol), blood glucose, and serum creatinine were measured at the time of SU assessment. Serum creatinine was quantified by spectrophotometry. Glomerular filtration rate (GFR) was estimated using the Chronic Kidney Disease Epidemiology Collaboration serum creatinine equation based on serum creatinine measurements [23].

#### 2.5.3. Symptoms & Palatability

After each intervention period, participants were asked to rate on a scale ranging from 0 to 9 the frequency with which they experienced the following symptoms during the preceding four weeks: hunger, bloating, constipation, diarrhea, excessive thirst, fatigue, headache, lightheadedness, or nausea. For this scale 0 represented “never occurred”, while 9 represented “experienced nearly every day.” Symptom responses were then dichotomized as never (a score of 0) versus any (a score of 1–9).

Participants were similarly asked about diet palatability: was the diet easy to follow, did you enjoy the diet, how likely are you to continue the diet after the study is over, was the quantity of food adequate, how often did you waste or store food, and how often did you supplement with non-study foods. For this scale, 0 represented “none of the time” or “least likely,” while 9 represent “all of the time” or “extremely likely.”

#### 2.5.4. Other Covariates

Age, sex, race, hypertension, diabetes, hyperlipidemia, and gout flare history were self-reported. Medications were self-reported at each visit.

### 2.6. Statistical Analysis

Means and standard deviations (SDs), medians and quartiles, or proportions were used to describe participant characteristics. According to the statistical analysis plan, prior to pooling periods for the primary outcome, an interaction by period was assessed to test for a diet-by-period interaction (see Supplement Figure S1). We observed opposing effects between periods 1 and 2, signifying the presence of carryover effects (the interaction term *p* = 0.11). As a result, we did not pool SU periods together. 

All contrasts (secondary outcomes, adherence, symptoms, and palatability) were performed comparing DDG to SDG. Comparisons were modeled using generalized estimating equation (GEE) regression with a Huber and White robust variance estimator, [24] which assumed an exchangeable working correlation matrix. Period 1 analyses were modeled using linear regression adjusted for baseline measurements, except for symptoms effects, which were not adjusted for baseline as symptoms were only assessed after each intervention period. 

In the DASH-Sodium trial (a controlled feeding study), we found that the DASH diet lowered SU by 1.3 mg/dL among adults with a baseline value ≥ 7 mg/dL [15]. Using a SD of 1.7 from DASH-Sodium (α = 0.05, power 0.8), we determined that we would need to crossover 41 adults to detect a difference of −0.75 mg/dL [25]. 

All analyses were performed with STATA version 15.0 (Stata Corporation, College Station, TX, US). Statistical significance was defined as two-sided *p* < 0.05 without Bonferroni adjustment.

## 3. Results

### 3.1. Baseline Characteristics

**Figure 1:**
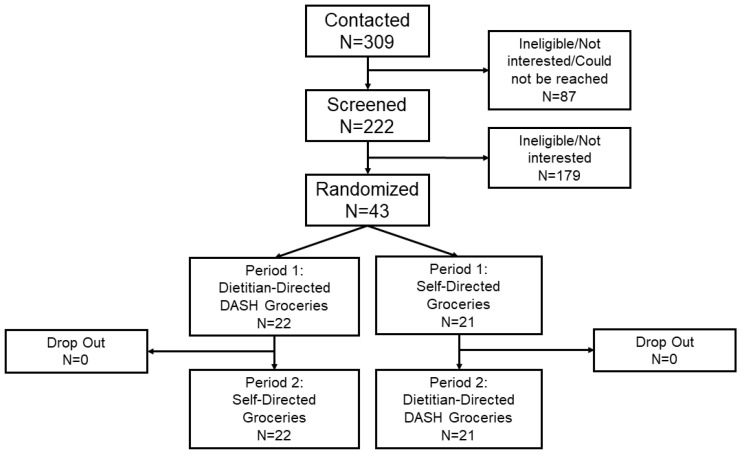
CONSORT Diagram.

Participant flow is displayed in Figure 1. Of 294 adults contacted, 43 people enrolled. The study population was of mean age 59.0 (SD, 12.1) years, 19% female, 49% black, and 63% had a history of hypertension (Table 2). Among the enrolled participants, 28% reported 1 gout attack while 67% reported >1 gout attack in the past year. Study participant characteristics were similar between intervention sequences.

### 3.2. Primary Outcome: Serum Urate

**Figure 2:**
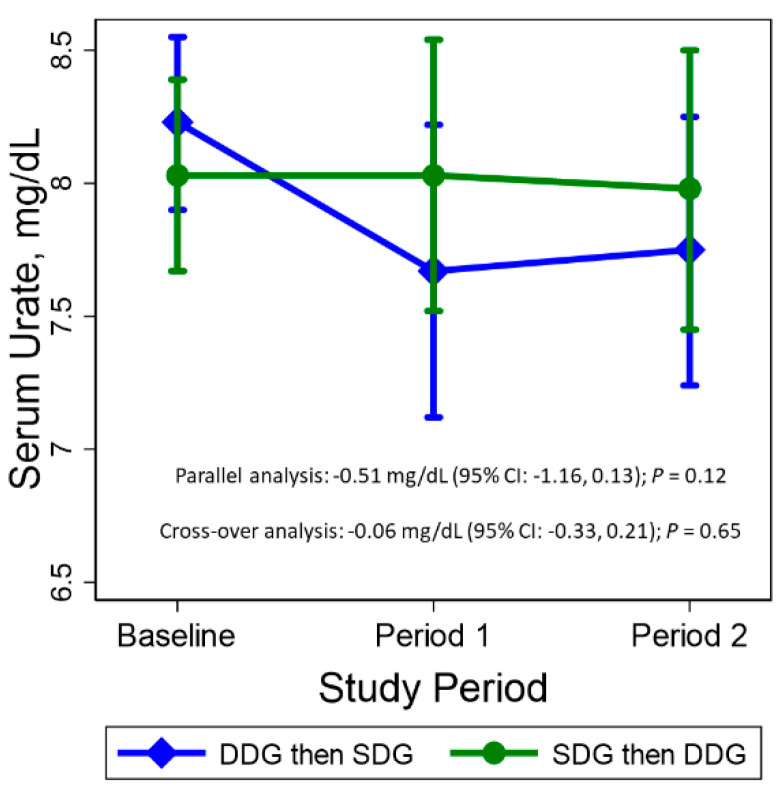
Mean serum urate by sequence assignment at baseline, period 1, and period 2. For the 22 assigned to the Dietitian-Directed DASH groceries (DDG) followed by the self-directed groceries (SDG), the mean serum urate at baseline, period 1, and period 2 was: 8.23 (SD, 0.74), 7.67 (SD, 1.25), and 7.75 (SD, 1.15) mg/dL, respectively. For the 21 assigned the self-directed groceries followed by the DASH groceries, the mean serum urate at baseline, period 1, and period 2 was: 8.03 (SD, 0.79), 8.03 (SD, 1.13), and 7.98 (SD, 1.15) mg/dL. The parallel analysis compared SU measured after period 1 between adults assigned DDG versus SDG. This comparison was adjusted for baseline. The crossover analysis compared DDG to SDG using SU measurements from both period 1 and period 2.

Among participants assigned to DDG during the first period, the baseline SU was 8.23 (SD, 0.74) mg/dL, while the baseline SU for those assigned SDG first was 8.03 (SD, 0.79) mg/dL (Figure 2). Compared to baseline, during Period 1 DDG lowered SU by 0.55 mg/dL (95% CI: −1.04, −0.07), while SDG had no effect on SU (0.0 mg/dL; 95% CI: −0.44, 0.44). However, after subjects crossed over to Period 2, the mean SU levels were virtually unchanged. At the end of the second period, compared to baseline, SDG reduced SU by 0.48 mg/dL (95% CI: −0.98, 0.01), while DDG reduced SU by 0.05 mg/dL (95% CI: −0.48, 0.38). The P for interaction by period was 0.11 (Supplement Table S1). 

There was no within-person difference in SU between the DDG and SDG periods when the planned crossover analysis was performed (difference −0.06 mg/dL; 95% CI: −0.33, 0.21). In a period 1 only analysis, compared to SDG, DDG reduced SU by 0.51 mg/dL (95% CI: −1.16, 0.13).

### 3.3. Other Outcomes

DDG non-significantly reduced SBP, DBP, and LDL-cholesterol (Table 3). DDG did not affect TUG test time, gout flares, and most of the pain parameters. The one exception was self-reported bodily pain, which was reduced by DDG (%-difference: −26.7%; 95% CI: −42.9, −5.9). 

### 3.4. Compliance Measures

Subjective and objective compliance measures are shown in Table 4. Self-reported fat, saturated fat, and cholesterol intake were reduced, while daily servings of fruit, vegetables, and beans significantly increased (all *p*-values ≤ 0.002). Furthermore, while there was no change in urate excretion, potassium, or urine pH, DDG significantly reduced urine sodium excretion (mean % difference = −22.4; 95% CI: −34.0, −8.6). 

### 3.5. Side Effects & Tolerability

During the DDG period, the DASH groceries were not associated with symptoms of hunger, bloating, diarrhea, thirst, fatigue, headache, lightheadedness, or nausea (Table 5). Furthermore, over 80% of participants reported that the DDG intervention was easy to follow or that they enjoyed the diet (Supplement Table S5). Notably, three-quarters of the participants reported they would likely continue the diet beyond the conclusion of the trial, whereas 88.4% reported that the quantity of food furnished in the trial was adequate. Only 20.9% reported wasting or storing food and only 7.1% reported supplementing with non-study foods.

### 3.6. Sensitivity Analysis: Period 1 Only (Parallel Effects)

We repeated analyses utilizing a parallel design, which did not meaningfully alter our findings (see Supplement Tables S2–S4) Section 3.1.

## 4. Discussion

Though relatively small in scale, this pilot study determined the overall feasibility of a community-based dietary intervention and suggests that providing dietitian-directed, DASH-patterned groceries may lower SU modestly over 4 weeks among gout patients not on urate-lowering therapy. Our intervention improved self-reported consumption of a DASH-pattern diet using a pragmatic approach, although implementation of a DASH-pattern diet was less stringent than in controlled feeding DASH trials. The DASH-patterned groceries were generally well-liked without side effects, with nearly three quarters of participants wanting to continue the diet after the trial’s conclusion. However, the open-label, behavior intervention, crossover design without a washout period likely led to a carryover effect in this trial. Definitive evaluation of the DASH diet as a treatment for gout would require a controlled feeding trial, ideally with a parallel design. 

Diet has been implicated in the pathogenesis of gout since antiquity [5]. This has long been attributed to the metabolism of purine-rich foods [26]. However, several clinical trials have recently demonstrated that dietary factors independent of purine metabolism, namely, vitamin C [9,16], dairy protein [11], and low glycemic index carbohydrates [27], are associated with SU reduction. These factors are inherent within the DASH diet, a diet proven to lower BP and LDL-cholesterol by emphasizing fruit, vegetables, low-fat dairy, and fiber, and reduced in saturated fat and cholesterol [28]. We have recently demonstrated that DASH adherence is associated with a lower risk of incident gout [14], and that DASH interventions lowered SU among adults without gout [15,16,29]. DIGO is one of the first trials to investigate a dietary intervention for adults with gout. As a pilot study, DIGO was designed to evaluate the feasibility and implementation of the DASH diet in gout patients rather than its conclusive efficacy. 

One of the challenges inherent to dietary interventions is how to achieve behavioral change in real world settings. In this pilot trial, we took the approach of allowing gout patients to choose foods according to their preference in a pattern concordant with the DASH diet. Similar to our previous dietary implementation trial among Urban African Americans [30], DIGO demonstrated the ability of DASH-patterned groceries to improve DASH adherence. However, based on prior controlled-feeding DASH trials among adults with a baseline SU ≥ 7 mg/dL, we predicted a treatment effect between 1–1.3 mg/dL [15,16]. It is likely that the provision of groceries only partly replaced the participants’ typical diets compared to a controlled-feeding study, which would underestimate the full dietary efficacy, contributing to the borderline SU reduction observed after the 4-week intervention period. To that end, an ideal trial to establish the efficacy of the DASH diet for urate reduction would require a controlled feeding study that provides all food consumed by participants, similar to the original DASH trials [12,13].

While the period-by-intervention interaction for a carryover effect was not statistically significant in our study, we likely lacked sufficient power. Regardless, intervention effects on SU did vary by period. As most participants found the DASH intervention easy to follow and reported being likely to continue the intervention after the study, without a washout period, participants initially assigned to the DDG could have continued eating a DASH diet during the SDG assignment. A mixed response of participants returning to their typical diet or continuing the DASH intervention may explain the modest increase in SU among this group during SDG. Alternatively, once there is a diet-induced reduction in SU, there might be a physiological lag in SU returning to baseline, particularly without controlled change in feeding. Moreover, in prior work we found that dietary effects from DASH plateaued after 8 weeks with the largest effect occurring within 4 weeks [31]. It is unknown whether the duration required for increasing SU is the same as for reducing SU such that these processes may not require equal time intervals. However, these hypotheses do not explain the near-absent effect observed by the SDG-to-DDG group. Further research is needed to disentangle these conflicting observations.

Our study has limitations inherent to behavioral intervention studies. Meals were not completely controlled, participants were unmasked, and compliance was promoted but not closely monitored. While participants did report dietary consumption that reflected the intended intervention, this may have been subject to Hawthorne effects. Moreover, the micronutrient content of the intervention was estimated and could vary further based on how food was prepared. Finally, the current pilot study was designed and the sample size powered for preliminary efficacy for SU, not our secondary outcomes. As such, null effects of the DASH diet, already proven to lower BP and LDL-cholesterol, should be interpreted cautiously. 

Our study has several strengths. First, to our knowledge, this trial is the first randomized trial of a dietary intervention among adults with gout. Our study demonstrates widespread interest among patients with gout for dietary interventions given our high recruitment and retention rates. Second, our study was racially diverse, and our outcomes rigorously ascertained with complete (100%) follow-up. Third, all grocery orders were delivered to our clinic for inspection by our teams of dietitians. This ensured high fidelity of the intervention and attests to the feasibility of this approach in future translational studies.

## 5. Conclusions

In conclusion, in this small scale pilot trial, the DASH-patterned grocery provision improved dietary adherence, and modestly lowered SU as well, despite apparent carryover. While DASH grocery delivery represents a promising intervention to promote healthy eating among community-dwelling adults, a definitive trial is needed to determine whether this approach reduces SU and CVD risk among individuals with gout.

## Figures and Tables

**Table 1:** Intervention goals and achieved servings or nutrients.

**Food Group**	**Servings/Day**	**Serving Size**
Grains	5–7	5.8 (2.2) oz equivalent
Vegetables	4	3.5 (0.9) cup equivalent
Fruits	4	3.0 (0.6) cup equivalent
Fat-free/low-fat dairy	2	2.3 (1.0) cup equivalent
Lean meats, poultry, fish	1–2	10.3 (2.7) oz equivalent
Nuts, seeds, legumes	<0.5	
**Nutrients**	**Original DASH**	Mean (SD) Ordered *
% Calories from fat	25.6	40.4 (7.6)
% Calories from saturated fat	7.0	8.5 (1.7)
% Calories from protein	17.9	18.7 (2.6)
% Calories from carbohydrates	56.5	40.9 (6.2)
Cholesterol (mg/day)	151	184.4 (65.7)
Potassium (mg/day)	4415	4110.9 (779.6)
Magnesium (mg/day)	480	534.2 (121.9)
Calcium (mg/day)	1265	1164.3 (366.4)
Sodium (mg/day)	2859	1466.9 (554.7)

* Mean (standard deviation, SD) values were estimated with the use of food analysis software (Food Processor SQL software, version 11.7.217; ESHA Research). These values represent raw/uncooked nutrients and do not account for nutrient losses during the cooking process.

**Table 2:** Characteristics of enrolled participants, overall and by their assigned order of intervention.

	Mean (SD), Median (25th, 75th), or %		
	Overall	Dietitian-Directed DASH Groceries in Period 1 (*N* = 22)	Self-Directed Groceries in Period 1 (*N* = 21)
Age, year	59.0 (12.1)	57.5 (13.3)	60.5 (10.7)
Female, %	19	18	19
Black, %	49	55	43
Body mass index, kg/m^2^	33.5 (6.8)	33.4 (6.4)	33.5 (7.5)
Systolic blood pressure, mm Hg	124.3 (14.8)	125.0 (17.0)	123.5 (12.4)
Diastolic blood pressure, mm Hg	73.8 (10.8)	74.9 (11.3)	72.6 (10.5)
Total cholesterol, mg/dL	191.4 (39.8)	187.0 (37.5)	196.0 (42.5)
Triglycerides, mg/dL	103 (77, 152)	89 (82, 147)	105 (75, 188)
HDL-cholesterol, mg/dL	52.7 (18.2)	53.6 (19.4)	51.7 (17.3)
LDL-cholesterol, mg/dL	112.2 (36.1)	109.0 (31.1)	115.5 (41.2)
Fasting blood glucose, mg/dL	97.9 (15.8)	93.0 (11.6)	103.0 (18.1)
eGFR, mL/min per 1.73 mm^2^	78.7 (16.1)	79.1 (16.6)	78.4 (16.0)
History of hypertension, %	63	50	76
History of high cholesterol *, %	45	43	48
History of diabetic condition **, %	20	10	30
Flare history			
No attacks past year	5	5	5
1 attack past year	28	23	33
>1 attacks past year	67	73	62
Diuretic use, %	28	27	29
Losartan use, %	21	18	24
Colchicine use, %	16	14	19
Non-steroidal anti-inflammatory (NSAID) use, %	14	14	14

Abbreviations: SD, standard deviation; * Note N for cholesterol was 42. ** Only 41 responded. Diabetes conditions included type 1 or type 2 diabetes, gestational diabetes, or a history of high blood sugar. Medication use is based on self-report at the first screening visit.3.2. Primary outcome: serum urate.

**Table 3:** Effects of Dietitian Delivered DASH versus Self-Directed Diet on Secondary Outcomes.

	Crossover Effect	
**Secondary CVD Endpoints**	**β (95% CI)**	***p***
Systolic blood pressure, mm Hg	−1.03 (−4.57, 2.52)	0.57
Diastolic blood pressure, mm Hg	−1.14 (−3.36, 1.09)	0.32
Body mass index, kg/m^2^	−0.15 (−0.39, 0.10)	0.24
HDL-cholesterol, mg/dL	−1.02 (−3.23, 1.19)	0.36
LDL-cholesterol, mg/dL	−4.99 (−12.32, 2.35)	0.18
Non-HDL-cholesterol, mg/dL	−6.23 (−13.67, 1.21)	0.10
Triglycerides, %	−5.0 (−12.9, 3.6)	0.25
Total cholesterol, mg/dL	−7.26 (−15.14, 0.62)	0.07
Fasting glucose, mg/dL	−3.05 (−7.03, 0.94)	0.13
eGFR, mL/min per 1.73 m^2^	1.14 (−1.05, 3.32)	0.31
**Pain & Physical Function**	**β (95% CI)**	***p***
Gout flares (Gaffo 2018 definition) *	0.66 (0.23, 1.89)	0.44
TUG test, seconds	−0.95 (−2.35, 0.46)	0.19
Pain with walking, %	−19.3 (−41.1, 10.7)	0.18
Pain with stair climbing, %	−24.1 (−43.2, 1.4)	0.06
Pain at night, %	−13.9 (−32.4, 9.7)	0.23
Pain at rest, %	−15.3 (−34.6, 9.9)	0.21
Pain with weight bearing, %	−15.3 (−35.5, 11.3)	0.23
Bodily pain, %	−26.7 (−42.9, −5.9)	0.01
Pain interfering with normal work, %	−12.1 (−33.6, 16.3)	0.37

Abbreviations: eGFR, estimated glomerular filtration rate; TUG, timed-up-and-go. All models are adjusted for baseline except gout flares, which was only assessed during follow-up. % indicates percent difference between periods. * Not assessed at baseline.

**Table 4:** Effects of Dietitian Delivered DASH (DDG) versus Self-Directed (SD) Diet on Compliance Measures.

	Crossover Effect	
**Self-reported food consumption**	**β (95% CI)**	***p***
Fat score, servings per day	−8.65 (−11.68, −5.62)	<0.001
Saturated fat, gm/day	−7.61 (−10.28, −4.95)	<0.001
Total fat, gm/day	−20.76 (−28.04, −13.49)	<0.001
Self-reported cholesterol, mg/day	−67.48 (−91.12, −43.84)	<0.001
Fruit & vegetable servings per day	3.12 (1.45, 4.78)	0.002
Fruit, vegetable, & bean servings per day	5.07 (2.92, 7.22)	<0.001
**Spot Urine**	**β (95% CI)**	***p***
Urate/creatinine, mg/mg	6.82 (−36.17, 49.81)	0.76
Sodium mmol/L, %	−22.4 (−34.0, −8.6)	0.002
Sodium mmol/L/creatinine mg/dL, %	−10.8 (−30.6, 14.6)	0.37
Potassium mmol/L, %	−4.4 (−19.4, 13.4)	0.61
Potassium mmol/L/creatinine mg/dL, %	9.3 (−3.3, 23.5)	0.15
Sodium mmol/L/potassium mmol/L, %	−18.4 (−34.5, 1.7)	0.07
Urine pH, %	2.1 (−1.8, 6.1)	0.30

All are adjusted for baseline; % is percent difference between periods.

**Table 5:** Effects of Dietitian-Delivered DASH (DDG) versus Self-Directed Groceries (SDG) on Symptoms.

Side Effects *	Crossover Effect	
	OR (95% CI)	*p*
Hunger	0.79 (0.38, 1.64)	0.53
Bloating	0.57 (0.32, 1.01)	0.054
Diarrhea	1.46 (0.64, 3.35)	0.37
Thirst	0.82 (0.42, 1.60)	0.57
Fatigue	0.68 (0.33, 1.39)	0.29
Headache	0.82 (0.41, 1.62)	0.57
Lightheadedness	1.00 (0.48, 2.06)	>0.99
Nausea	0.39 (0.10, 1.49)	0.17

* These are not adjusted for baseline as side effects were only assessed after period 1 and period 2. Side effects were based on a 0–9 response on a Likert scale, ranking the frequency of each symptom with 0 being never and 9 being “nearly every day.” These effects were treated as a dichotomous variable with 0 being never and 1–9 treated as any symptoms.

## Data Availability

Requests for data may be directed to the corresponding author and are subject to institutional data use agreements.

## Supplementary Materials

The following are available online at https://www.mdpi.com/2072-6643/13/2/538/s1, Figure S1: Interaction plot; Table S1: Change in serum urate from baseline by assignment; Table S2: Effects of DASH versus self-directed diet on serum urate and secondary outcomes (parallel effect); Table S3: Effects of DASH versus self-directed diet on compliance measures (parallel effect, Period 1 only); Table S4: Effects of DASH versus self-directed diet on symptoms (parallel effect, Period 1 only); Table S5: Palatability questions, reported on a 0–9 Likert scale; Supplement Material S1: Qualitative description log of grocery quality for 43 participants, each with 4 weeks of $105 work of groceries/week.

## Abbreviation

BMI, body mass index; CI, confidence interval; DASH, Dietary Approaches to Stop Hypertension; DIGO, The Dietary Approaches to Stop Hypertension (DASH) Diet Effects on Serum Urate (SU) in Adults with Hyperuricemia and Gout.
