# Supplementary material for: A Randomized Pilot Study of DASH Patterned Groceries on Serum Urate in Individuals with Gout
Source: Nutrients. 2021 Feb 7;13(2):538. doi: 10.3390/nu13020538 (PMC7914968; doi:10.3390/nu13020538)
Supplement: Supplementary file 1 [file nutrients-13-00538-s001.pdf]

Supplement Material

Supplement Figure SF1. Interaction plot

Supplement Table ST1. Change in serum urate from baseline by assignment

Supplement Table ST2. Effects of DASH versus self-directed diet on serum urate and secondary outcomes (parallel effect)

Supplement Table ST3. Effects of DASH versus self-directed diet on compliance measures (parallel effect, Period 1 only)

Supplement Table ST4. Effects of DASH versus self-directed diet on symptoms (parallel effect, Period 1 only)

Supplement Table ST5. Palatability questions, reported on a 0-9 Likert scale

Supplement Material SM1. Qualitative description log of grocery quality for 43 participants, each with 4 weeks of $105 work of groceries/week

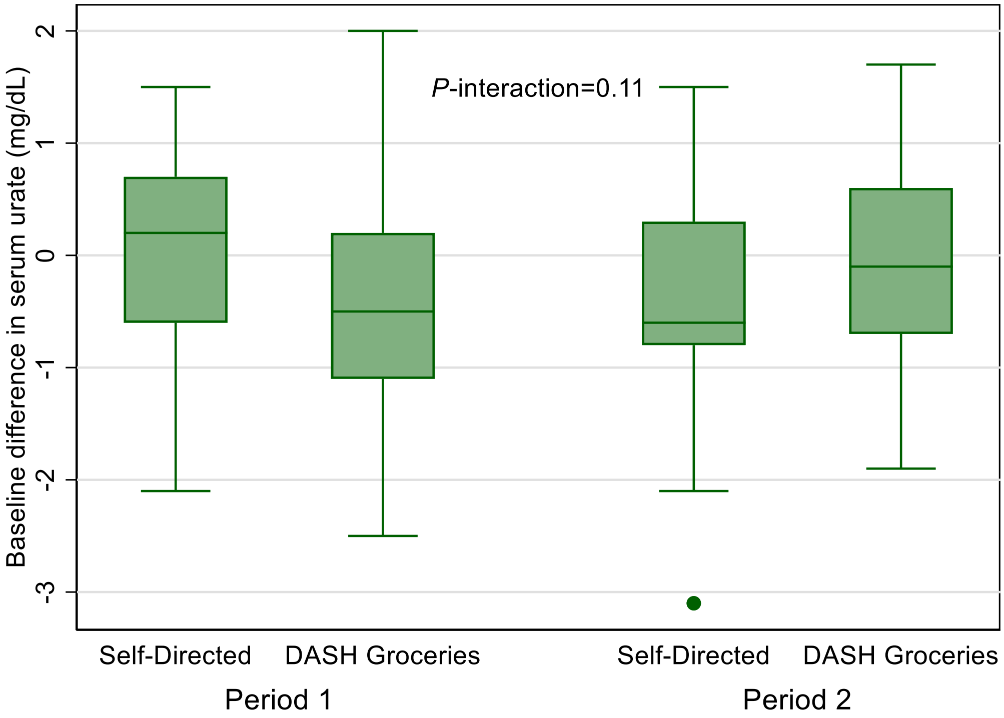

Supplement SF1. Interaction plot of median and 25th to 75th percentiles of the difference in serum urate (mg/dL) from baseline according to intervention and study period. Carryover was assessed using an interaction of intervention by period adjusted for baseline serum urate level. There was a notable difference in the direction of the effect between Dietitian-Directed DASH groceries and self-directed groceries with *P*-interaction=0.11. Based on the non-significant interaction term, we pooled the trial periods together.

Supplement Table ST1. Change in serum urate from baseline by assignment

**Mean Differences (95% CI)**	**N**	**Period 1**	**Period 2**
**Overall**	43	-0.28 (-0.60, 0.03)	-0.27 (-0.59, 0.04)
**DDG followed by SDG**	22	-0.55 (-1.04, -0.07)	-0.48 (-0.98, 0.01)
**SDG followed by DDG**	21	0.00 (-0.44, 0.44)	-0.05 (-0.48, 0.38)

Note the period-by-intervention coefficient was 0.11. DDG represents dietitian directed groceries, SDG represents self-directed groceries. Note that “DDG followed by SDG” corresponds to the green (circle) line in Figure 1 and “SDG followed by DDG” corresponds to the blue (diamond) line in Figure 1.

Supplement Table ST2. Effects of DASH (DDG) versus Self-Directed (SD) Diet on Secondary Outcomes (Parallel Effect)

	Period 1 (Parallel Effect)	
**Secondary CVD endpoints**	**β (95% CI)**	***P***
Systolic blood pressure, mm Hg	1.60 (-5.01, 8.21)	0.63
Diastolic blood pressure, mm Hg	-0.92 (-5.60, 3.75)	0.69
Body mass index, kg/m^2	-0.38 (-0.77, 0.01)	0.05
HDL-cholesterol, mg/dL	-2.09 (-7.44, 3.26)	0.43
LDL-cholesterol, mg/dL	-2.12 (-12.95, 8.71)	0.69
Non-HDL-cholesterol, mg/dL	-2.37 (-13.27, 8.52)	0.66
Triglycerides, %	-9.1 (-23.5, 7.9)	0.27
Total cholesterol, mg/dL	-5.43 (-18.11, 7.25)	0.39
Fasting glucose, mg/dL	3.02 (-7.09,13.13)	0.55
eGFR, mL/min per 1.73 m^2	1.48 (-4.25, 7.21)	0.60
**Physical Function & Pain**	**β (95% CI)**	***P***
Gout flares (Gaffo 2018 definition)*	1.25 (0.29, 5.47)	0.77
TUG test, seconds	0.71 (-0.69, 2.11)	0.31
Pain with walking, %	-24.8 (-55.6, 27.2)	0.28
Pain with stair climbing, %	-35.9 (-61.5, 6.6)	0.08
Pain at night, %	-28.3 (-55.8, 16.2)	0.17
Pain at rest, %	-31.2 (-56.1, 7.9)	0.10
Pain with weight bearing, %	-24.8 (-55.5, 26.9)	0.28
Bodily pain, %	-32.4 (-55.6, 3.0)	0.07
Pain interfering with normal work, %	0.6 (-36.6, 59.6)	0.98

All are adjusted for baseline except gout flares, which was only assessed during follow-up.

*Not assessed at baseline.

Supplement Table ST3. Effects of Dietitian-Directed DASH Groceries versus Self-Directed Diet on Compliance Measures according to the Parallel Design (Period 1 Only)

	Period 1 (Parallel Effect)	
**Self-reported food consumption**	**β (95% CI)**	***P***
Fat score, servings per day	-9.05 (-13.75,-4.35)	<0.001
Saturated fat, gm/day	-7.96 (-12.03,-3.89)	<0.001
Total fat, gm/day	-21.66 (-33.21,-10.10)	<0.001
Self-reported cholesterol, mg/day	-69.33 (-106.20,-32.46)	<0.001
Fruit & vegetable servings per day	3.72 (1.39, 6.04)	0.0025
Fruit, vegetable, & bean servings per day	6.79 (3.64, 9.94)	<0.001
**Spot Urine**	**β (95% CI)**	***P***
Urate/creatinine, mg/mg	-4.39 (-82.33,73.55)	0.91
Sodium mmol/L, %	-10.1 (-34.5, 23.3)	0.50
Sodium mmol/l/creatinine mg/dl, %	7.3 (-28.4, 60.7)	0.73
Potassium mmol/L, %	3.3 (-21.9, 36.7)	0.82
Potassium mmol/L/creatinine mg/dl, %	21.8 (-1.2, 50.2)	0.06
Sodium mmol/L/potassium mmol/L, %	-8.4 (-34.4, 27.9)	0.60
Urine pH, %	4.7 (-2.4, 12.3)	0.19

All are adjusted for baseline

Supplement Table ST4. Effects of DASH versus Self-Directed Diet on Symptoms Experience the Preceding Four Weeks according to the Parallel Design (Period 1 Only)

	**Period 1 (Parallel Effect)**	
**Side effects***	**OR (95% CI)**	***P***
Hungry	0.57 (0.14, 2.34)	0.44
Bloating	1.60 (0.42, 6.03)	0.49
Diarrhea	1.31 (0.34, 5.05)	0.69
Thirst	0.53 (0.16, 1.82)	0.32
Fatigue	0.56 (0.16, 1.97)	0.36
Headache	1.70 (0.50, 5.83)	0.40
Lightheadedness	1.31 (0.34, 5.05)	0.69
Nausea**	-	-

*These are not adjusted for baseline as side effects were only assessed after periods 1 and periods 2. Side effects were based on a 0-9 response on a Likert scale, ranking the frequency of each symptom with 0 being never and 9 being "nearly every day." These outcomes were treated as a dichotomous variable with 0 being never and 1-9 treated as any symptoms.

**There were 5 people (all in the self-directed group) with symptoms of nausea after period 1, but none with nausea in the DASH-Grocery intervention

Supplement Table ST5. Palatability Questions, reported on a 0-9 Likert scale according to period.

**Period 1, N=22**			
**Question**	**Mean**	**% 0-2**	**% 7-9**
Easy to follow	7.4	4.5	77.3
Enjoyed the diet	7.7	4.5	81.8
Likely to continue diet	7.4	0.0	72.7
Quantity of food was adequate	8.0	0.0	86.4
Wasted or stored food	4.5	27.3	27.3
Supplemented with non-study foods	1.7	72.7	4.5
**Whole cohort, N=43**			
**Question**	**Mean**	**% 0-2**	**% 7-9**
Easy to follow	7.6	4.7	81.4
Enjoyed the diet	7.8	2.3	81.4
Likely to continue diet	7.5	0.0	74.4
Quantity of food was adequate	8.2	0.0	88.4
Wasted or stored food	3.7	41.9	20.9
Supplemented with non-study foods	1.7	76.2	7.1

Note: 0 represented “none of the time” or “least likely,” while 9 represent “all of the time” or “extremely likely.”

Supplement Material SM1. Intervention Instructions

Eating Guidelines While on the Food Delivery Section of the Study

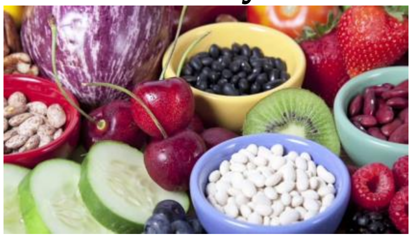

In this study you will be receiving some fruits, vegetables, nuts, beans, grains, lean meat and low fat dairy products as party of a healthy diet. This will provide you with a basic daily eating plan but it may not completely meet your dietary needs for the day. When deciding with foods to eat to compliment the foods you were given, consider the following:

1) Drink plenty of water throughout the day.
2) Avoid excessive alcohol intake.
3) Avoid a kind of sugar called fructose – this is found in sodas and many juices and some other sweets such as candy, ice cream, store bought baked goods and processed foods and some cereals.
4) Avoid red meat, organ meat (like liver and kidney) and avoid shellfish like
shrimp, lobster, crab, anchovies and sardines.
5) You can enjoy the fruits, vegetables, nuts, beans and grains that will be provided for you in the study. You can eat more of those same types of food if desired.
6) Even though it is helpful to watch your weight, **DO NOT** go on strict weight loss diets.
7) Your healthy balanced diet can include low fat or no fat dairy products such as low fat milk and cottage cheese.

8) Your healthy balanced diet can include moderate amounts of lean chicken and fish.
9) Don’t make drastic changes in your salt intake.

Supplement Material SM2. General instruction provided to participants throughout the study.

GENERAL DIETARY INFORMATION

Throughout the DIGO Study there are some basic recommendations to guide you in better food and lifestyle choices while dealing with gout.

1) **Drink adequate water throughout the day and don't allow yourself to become dehydrated.**
2) **Avoid strict weight loss diets.**
3) **Avoid excessive alcohol intake**
4) **Avoid sugar sweetened beverages.**
5) **Don't make drastic changes in your salt intake.**
6) **Limit meat.**

Supplement Material SM3. Instructions related to food pick up.

SAFE FOODS TO GO

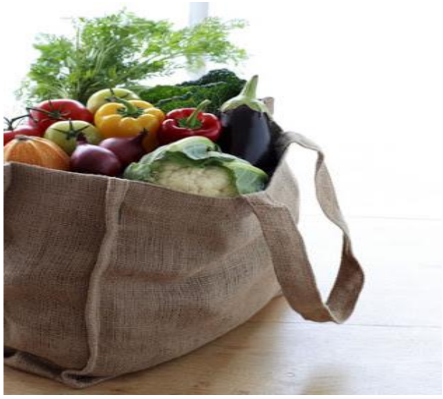

You will be picking up food at ProHealth once a week for 4 weeks and we want to be sure you transport your food safely. You will be receiving orders of fruits and vegetables that could be fresh, canned or frozen, other grocery items such as cereal, bread or grains, and fresh meat and dairy products. Many of these products are perishable and care needs to be taken in their transport from ProHealth home and once you arrive in your home. There are several things to consider:

1. You will be receiving enough food for a week, so please be sure you have room in your refrigerator for all the groceries.
2. Do not transport food in the trunk of your car. Be sure to transport food in the main part of your car where it is air conditioned.

3. Plan your food pick-up so that you can go right home or to a location where there is a refrigerator and freezer if needed, and get your food in a safe storage temperature as soon as possible. We will have ice packs available if needed.
4. If you suspect some of your study foods may be spoiled,
   Do not eat the perishable food items contained in the package if you suspect that they may be spoiled. You should immediately notify us and follow our instructions on what to do next. We may provide you a

replacement food, or ask if you can purchase a similar food and give us the bill for the purchase.

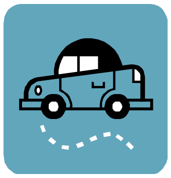

SAFELY ENJOY YOUR TO GO FOODS

Supplement Material SM4. Qualitative description log of grocery quality for 43 participants, each with 4 weeks of $105 work of groceries/week

Participant 1: Week 1 Green Beans Half Brown

Participant 2: Week 2 Cucumbers – Spoiled

Participant 3: Week 2 No milk until Wednesday, got produce at store. Week 3 Salmon out of date. Week 4 Kiwi – one bad.

Participant 4: Week 1 – questions on potatoes, sweet potatoes (amount excessive), garlic, beets and ginger. Week 3 – Ordered Fugi apples but got Granny Smith, Feta Cheese out of stock.

Participant 5: Week 3 – Tomato Paste – 2 came in smashed.

Participant 6: Week 1 – Ordered Spring mix and got Spinach Salad. Week 2 – Ordered Thin Whole Grain Bread and got Whole wheat. Week 4 – Oranges – 1 bad out of 6.

Participant 7: Week 3 – Got 2 salad mixes – ordered 1

Participant 8: Week 3 - I forgot to give frozen vegetables.

Participant 9: Week 1 – 2% Milk didn't come. Week 2 – Blueberries ordered 2 didn't get. Week 3 – Ordered 2 small string cheese – out of stock – got one large string cheese.

Participant 10: Week 2 – Ground Turkey okay, but expiration date same as delivery date.

Participant: Week 1 – Olive oil not available – got at store. Tomatoes not sent. Got at store. Ordered one baby carrots, got 2. Week 3: Strawberries not sent. Olive oil not available – got all at store.

Participant: Week 2 – Ordered 2 blueberries, didn't get. Beets out of stock – got from store. Week 3 – 5/7/2019 expiration date on chicken appeared to be 3/2019. Not given.

Participant: Week 3 – Ordered 2 spinach and got 3.

Participant: Week 1 – Brown ride – didn't get. Clementines and strawberries – ordered 1 got 2.

Participant: Week 2 – 6/3/2019 – expiration on chicken 6/2/2019.

Participant: Weeks 1 and 2 – Egg Beaters not in stock.

Participant: Week 1 – only one tomato paste available. Week 4_– Almond Milk fell out of box – replaced. Yogurt ordered 12 – got 8 and 1 out of date. Blueberries – mold, Strawberries – soft on top.

Participant: Week 2 – Garbonzo Beans – on can dented. 8 yogurts – 1 smashed. Week 3 – 8 yogurts – 1 smashed.

Participant: Week 1 – 6/3/2019 – chicken dated 6/2/2019. Week 4 – Smart Balance not in stock.

Participant: Week 1 – Missing tomatoes. Week 2 – Didn't get peanuts. Week 3 – Lettuce ordered 2 got one. Tomatoes moldy, Blueberries – one smashed.

Participant: Week 2 – didn't get sweet potatoes.

Additional Field Notes from our Research Dietitian:

This is a rough review of order sheets for the study. There were many issues associated with food delivery that should be incorporated into the discussion of the intervention. These include:

1) Issues with the order site and need to reserve a time slot early in the day or lose the only time slot that was slated for delivery during our work day hours. More delivery times became available later in the study.

2) When we first started to order, they were not set up to meet our demands. We often could only order a few of something like salmon, and had to purchase some at the store if we had more participants wanting it.

3) They were not set up for a company so they could not break orders up into individuals in a same day. Later they could.

4) Initially the delivery was all piled up on the porch first thing in the morning, and packages were falling apart and food was not packaged safely. I complained to them when raw chicken was in the same bag with salad greens. We were given refunds when I complained.

5) Ordering became better as time went by, but there were still times when products were out of stock, there was a limit to how many you could purchase, bananas and salad and milk could not be ordered or Thursday or Friday for Monday. An updated order had to be placed on the weekend. Milk was often not available until Tuesday.

6) We had several times of concern about the products and were given refunds, but we had to go to a site and go on a chat box. The procedure to make corrections is not very consumer friendly.

7) Week of November 20, 2018 – Called participants to have them dispose of romaine lettuce, spring mix including romaine lettuce and Jenni O Ground Turkey – due to recalls on those products.

8) Week of June, 11, 2019 – Ordered food from Whole Foods for everyone that week. Amazon shut down. Got bread and nuts from store. Products from Whole foods were delivered in a timely fashion, packaged well and good items.
